# Molecular characteristics, immune evasion, and impact of SARS-CoV-2 variants

**DOI:** 10.1038/s41392-022-01039-2

**Published:** 2022-06-28

**Authors:** Cong Sun, Chu Xie, Guo-Long Bu, Lan-Yi Zhong, Mu-Sheng Zeng

**Affiliations:** 1 State Key Laboratory of Oncology in South China, Collaborative Innovation Center for Cancer Medicine, Department of Experimental Research, Sun Yat-sen University Cancer Center, Sun Yat-sen University, 510060 Guangzhou, China; 2 Guangdong-Hong Kong Joint Laboratory for RNA Medicine, 510060 Guangzhou, China

**Keywords:** Vaccines, Infectious diseases, Infectious diseases

## Abstract

The persistent COVID-19 pandemic since 2020 has brought an enormous public health burden to the global society and is accompanied by various evolution of the virus genome. The consistently emerging SARS-CoV-2 variants harboring critical mutations impact the molecular characteristics of viral proteins and display heterogeneous behaviors in immune evasion, transmissibility, and the clinical manifestation during infection, which differ each strain and endow them with distinguished features during populational spread. Several SARS-CoV-2 variants, identified as Variants of Concern (VOC) by the World Health Organization, challenged global efforts on COVID-19 control due to the rapid worldwide spread and enhanced immune evasion from current antibodies and vaccines. Moreover, the recent Omicron variant even exacerbated the global anxiety in the continuous pandemic. Its significant evasion from current medical treatment and disease control even highlights the necessity of combinatory investigation of the mutational pattern and influence of the mutations on viral dynamics against populational immunity, which would greatly facilitate drug and vaccine development and benefit the global public health policymaking. Hence in this review, we summarized the molecular characteristics, immune evasion, and impacts of the SARS-CoV-2 variants and focused on the parallel comparison of different variants in mutational profile, transmissibility and tropism alteration, treatment effectiveness, and clinical manifestations, in order to provide a comprehensive landscape for SARS-CoV-2 variant research.

## Introduction

The COVID-19 pandemic has lasted for over 2 years and caused over 6 million death cases.^1^ A wide variety of SARS-CoV-2 variants emerged during its persistence and displayed evolving adaptation to global populational immunity,^2–5^ leading to rapid worldwide spread and heterogenous escape from available therapeutic drugs and vaccines.^6–9^ The mutations harbored in the genome of SARS-CoV-2 variants have a significant impact on viral protein structures, function, and immunogenicity, which was strongly associated with the immunological response and clinical outcome in humans.^10–13^

This review systematically describes the evolutionary and molecular characteristics of SARS-CoV-2 variants and summarizes the mutational impact on the critical viral proteins. Then it comprehensively describes the landscape of immune evasion of various critical variants from the currently approved antibody, small antiviral molecules, and vaccines. Lastly, it describes the epidemiological profile of SARS-CoV-2 variants and overview the different critical strains’ changes in infectivity, host tropism, and clinical manifestation and outcome. Detailed datasets for the parameterized depiction of the difference between SARS-CoV-2 variants in molecular characteristics, immune evasion, and clinical impact are also provided.

## Molecular characteristics of sequence and the encoded proteins of SARS-CoV-2 variants

### The genomic evolution of SARS-CoV-2

Since the emergency of SARS-CoV-2,^14–17^ its viral genome has been under constant and rapid mutation to adapt host system.^18,19^ Like other RNA virus,^20–25^ a high mutation rate benefits the emergence of novel variants with a significant change in viral phenotypes.^20,26^ Therefore, the global scientific community endeavors to construct systematic tracking systems of SARS-CoV-2 mutations and identified the clade with a genetically close relationship.^27^

**Fig. 1:**
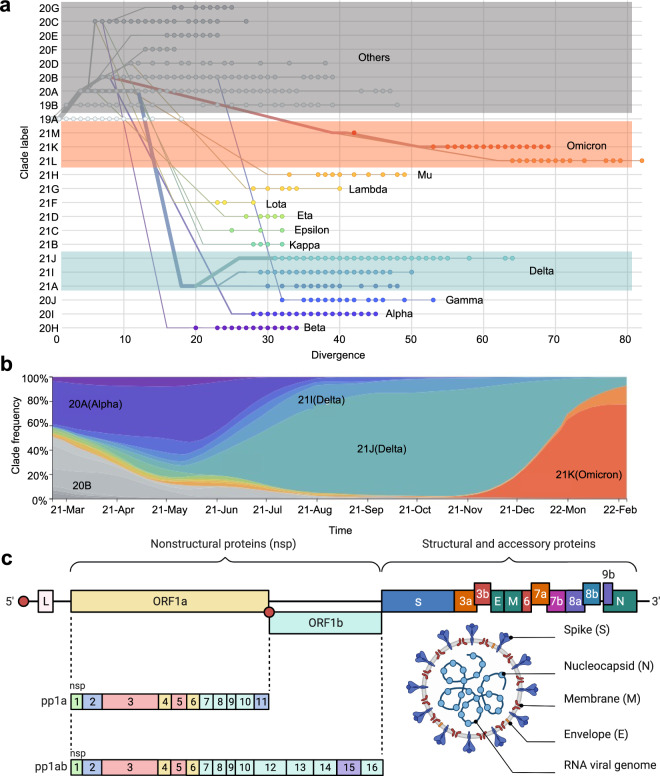
SARS-CoV-2 evolution, prevalence, and genome architecture. **a** Phylogenetic analysis of sequence divergence of SARS-CoV-2 circulating variants based on clade classification in February 2022. The WHO labeling of clades is marked besides. **b** Sequential frequency of major clades of SARS-CoV-2 variants from April 2021 to February 2022. **c** Linear genome architecture of encoded viral protein and structural overview of SARS-CoV-2. The phylogenetic analysis and sequential frequency data come from the Nextstrain GISAID database (https://nextstrain.org/ncov/gisaid/global), and figures in related (**a**, **b**) are generated under the CC-BY 4.0 permission. BioRender is used to generate the structure diagram of SARS-CoV-2 virus in Fig. 1c

The phylogenetic classification is widely used as a fundamental method for emergent SARS-CoV-2 strain classification in the clade-nomenclature system (terming the major strain as clade code such as GR) by Global Initiative of Sharing All Influenza Data (GISAID)^28^ or NextStrain^29^ or Pango lineage system (terming the major strain as letter and number with point interval such as B.1.1.7) by Pango Network^30^ (Fig. 1a). However, with the rapid increase in submitted sequence to the genomic database and wider observation of sequential distribution in the infected population, a more compact naming system for the critical variants was demanded to guide global anti-virus policy. Therefore World Health Organization (WHO) proposed using the Greek alphabet to name the critical SARS-CoV-2 clades or Pango lineages and raised the concept of Variant of Concern (VOCs) and Variants of Interest (VOIs) as a larger dynamic classification.^17^ Our review used the WHO naming system to indicate the strains in representing both sequence identity and their impact on disease control.

Early 2020 has witnessed the emergence of the first widely reported spike mutation of SARS-CoV-2, D614G.^31–36^ In December 2020, the Alpha variant (B.1.1.7) harboring another critical mutation N501Y^37,38^ in spike protein, initially expanded in the southeast of England, soon became the first globally distributed VOC (Fig. 1b).^39–41^ Later the Beta variant (B.1.351) was found in South Africa and manifested a rapid domestic distribution to an over 80% prevalence.^42,43^ One month later, the Gamma variant (P.1) was reported in Brazil, and the travelers arriving in Japan from Brazil.^44,45^ Delta variant (B.1.617.2) was first detected in India in May 2021 and rapidly became the dominant variant worldwide by late 2021, while some sub-clade of Delta variant displayed a unique penchant in epidemic areas, such as Clade 20I (Delta) in some parts of Asia.^41,46,47^ Delta-dominant epidemic lasted quite long in the world until Omicron (B.1.1.529) in November 2021, which was first reported in South Africa,^48,49^ and soon in Chinese Hong Kong.^50^ Since its discovery, Omicron rapidly displaced Delta and became the major variant worldwide.^48,51–54^ By the time of 31 March 2022, only variants Alpha (B.1.1.7), Beta (B.1.351), Gamma (P.1), Delta (B.1.617.2), and Omicron (B.1.1.529) were labeled as Variant of Concern (VOCs), within which only Delta and Omicron were recognized as currently circulating VOCs.^50,55–61^

As the SARS-CoV-2 RNA genome encoded a set of structural and non-structural proteins (Fig. 1c),^62–65^ the mutations in these proteins lead to various molecular alterations in protein characteristics, shaping the difference between variant to variant.^66^

### SARS-CoV-2 spike protein

**Fig. 2:**
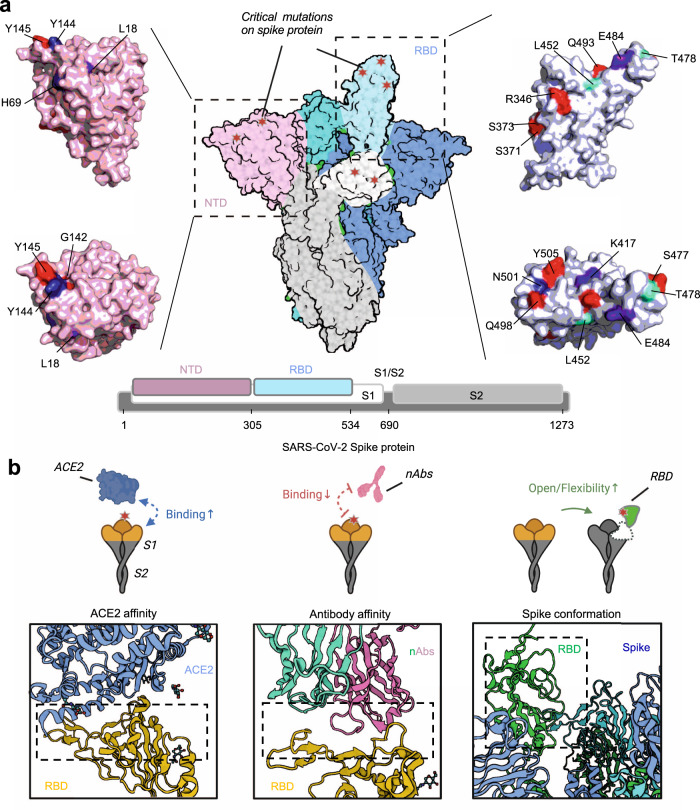
Mutations and their effect on SARS-CoV-2 spike protein. **a** Structural overview of SARS-CoV-2 spike protein and its subdomain RBD and NTD. Mutations from variants were marked beside the colored surface. **b** Patterns of mutational impact on the spike protein. Mutations affect the spike affinity to ACE2 and neutralizing antibodies (nAbs) and influence the spike protein conformations. BioRender is used to generate the structural presentation and cartoon models. Pymol is used to generate the surface model of RBD or NTD region. 6VSB, 7CM4, and 6M0J structures are retrieved from PDB database

**Fig. 3:**
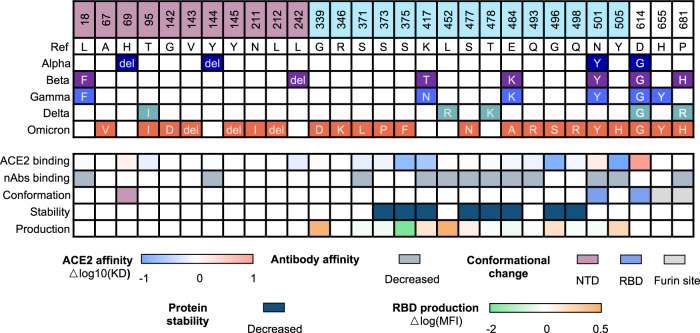
Heatmap of the mutation site and the mutational impact of SARS-CoV-2 VOCs on the spike protein characteristics. The mutations of VOCs vary from strain to strain and could exert multiple impacts on the protein characteristics of spike protein, including the ACE2 binding, antibody affinity, protein conformation, stability, and productivity. Quantitative results were recorded on the influence of ACE2 affinity and RBD production, and qualitative results were recorded on antibody affinity, conformational change, and protein stability. Details for each mutation can be found in Supplementary Table 1

The SARS-CoV-2 spike protein, as the major structural protein, is embedded in the SARS-CoV-2 viral membrane in homo-trimeric form and recognizes human ACE2 as a receptor for viral entry.^65,67–70^ It consists of two subunits, S1 and S2, cleaved by host furin.^65,71–76^ The distal S1 subunit contains two important regions, RBD (receptor binding domain) and NTD (N-terminal domain),^77^ and the RBD acts as the binding region for ACE2,^77–79^ making it the most critical target affecting virus-host interaction and vulnerable site to antibody neutralization^80–84^(Fig. 2a). Currently, most neutralizing antibodies or vaccines are developed to target the RBD to block or inhibit viral infection.^80,84–93^ Furthermore, the binding with ACE2 of RBD requires conformational adaptation, and an easier transition from “closed” to “open” conformation of spike protein benefits the viral infection.^32,94–96^ Therefore, mutations in the spike protein of SARS-CoV-2 variants could significantly influence the structure of the spike protein conformation and further the interaction with ACE2 or neutralizing antibodies^32,95,97–100^ (Figs. 2b and  3).

#### Impact on ACE2 binding

In vitro binding experiments have shown that SARS-CoV-2 bound to human ACE2 with an affinity of about 10 nM, which was 10–20-folds higher than SARS-CoV,^79,87,101,102^ which is a potential reason for the higher infection rate of SARS-CoV-2.^103^ The residues of RBD directly participated in ACE2 binding^79^ are K417, Y449, Y453, N487, Y489, G496, T500, G502, Y505, L455, F456, F486, Q493, N501, Q498, and the mutations at or beside these sites may directly impact the interaction with ACE2.^103^ The N501Y is one of the most common mutations at spike protein and could be found in Alpha, Beta, Gamma, and Omicron. Various studies have demonstrated that this mutation enhances the binding with human ACE2 through the extra introduction of π-π packing between RBD Y501 and ACE2 Y41.^103,104^ Likewise, mutation E484K from Beta and Gamma variant or L452R and T478K from Delta variant was also reported to increase the affinity with ACE2 by mutational scanning or computational analysis.^105–108^ However, not all the mutations at RBD from variants benefited ACE2 binding. Studies showed that mutation at K417 from Beta and Gamma variants could impair the RBD binding with ACE2.^103–105^ More mutations from Omicron were yet to be studied on their impact on ACE2 binding affinity, despite some of them being quite far from the interface with ACE2.^53,55,58,109,110^ SARS-CoV-2 evolved to adapt to the host, leading to widespread circulation among animals while still retaining its ability to efficiently utilize human ACE2 for entry, thus allowing for transmission of the virus back into humans.^99,111^

#### Impact on antibody binding

The spike protein could elicit a protective antibody against viral infection through the key regions mediating viral entry and fusion. Therefore, there were early explorations of using anti-SARS-CoV spike antibodies to neutralize SARS-CoV-2 due to the high sequence similarity.^81,87^ Unfortunately, none of them (m396, S230, 80R, CR3014) manifested obviously neutralization activity against SARS-CoV-2 infection,^67,87,102,112^ and these results provided primary evidence of antibody escape caused by the amino acid substitution in antigen.^79,101^ As more antibodies targeting SARS-CoV-2 spike or RBD were isolated or developed, many of them with potent neutralizing capability and clinical perspective were reported and investigated in-depth with biological and structural experiments.^79,106,113–120^

For mutations located on RBD, a structural study revealed that K417 mutation from Beta, Gamma, and Omicron reduced antibody binding to spike protein of C682, C614, and C653,^121,122^ E484K from Beta and Gamma diminished binding of C602, C627, C628, C653, C643 and C6710,^105,121,123,124^ and N501Y from Alpha, Beta, Gamma and Omicron diminished binding of C613, C628, C663, and C670.^121^ It was also revealed that L452R from Delta variant reduced DH1041 binding to spike protein.^124–126^ Another mutational analysis evaluating the overall mutation sets from SARS-CoV-2 variants based on computational analysis and in vitro experiment also indicated that currently approved antibodies, including REGN-10933 (Casirivimab), REGN-10987 (Imdevimab), and CT-P59 (Regdanvimab) displayed decreased affinity with spike proteins from all VOCs.^60,127^

For mutations not located on RBD, L18F in Gamma, T19R in Delta and Omicron, D80A in Beta, G142-/D, and Y144- in Omicron reduced S2L28, S2X28, S2M28, S2X333 and 4A8 (PDB:7C2L) binding to spike protein.^115,128^ The deletion of amino acid residues 241~243 in NTD of the Beta variant nearly abolished the binding of 4A8, an antibody targeting the NTD domain.^107,115^

#### Impact on protein conformation, yield, and stability

The conformation of spike protein also determined the efficacy of ACE2 binding, as the spike protein had two conformations, “open” and “close,” in the representation of the sub-structural arrangement of RBD as “up” or “down”. Only spike protein with at least one RBD in “up” conformation could be bound by ACE2.^94^ Therefore, the mutational impact on the spike conformation would also influence the spike protein binding with ACE2. Some studies revealed that N501Y and D614G could facilitate the transition of spike protein from “closed” to “open“.^31,32,34,95,100,129^ Furthermore, mutations at H655 and P681 were reported to increase the cleavage efficiency of spike protein at furin site to promote viral-cell membrane fusion.^104,130–133^ As for the NTD domain, it was reported that H69del/V70del in the Omicron and Alpha variant resulted in the contraction of NTD and lead to a tighter NTD configuration.^114–116^

Moreover, the stability and yield of spike protein upon the viral membrane would also influence the overall infectivity, as associated with the availability of spike protein for viral entry.^34,36^ A series of mutations were associated with the instability of spike protein,^55,57,134^ while one study made a parallel analysis of the RBD productivity under various mutations and found that most prevalent mutations would facilitate the yield of RBD.^103^ These results provided another perspective to analyze the mutational impact on the spike protein.

### SARS-CoV-2 structural proteins beyond spike

Except from the spike, there are other three structural proteins: Envelop (E), Membrane (M), and nucleocapsid (N), encoded by ORF4, ORF5, and ORF9.^135–138^ E and M majorly participate in virion assembly,^136,139^ while N forms the viral capsid structure associated with viral RNA and facilitates genome packaging. Despite not participating in the initiation of viral infection, these proteins had a significant role in viral replication, assembly, and release. Their mutations could also influence viral activity.^137,140–143^

Compared with E or M, much more mutations in N protein were observed,^56,144,145^ and Omicron had the most abundant mutations, including P13L, E31del, R32del, S33del, and S413R as unique mutations, and 203K (Alpha, Gamma, Delta, and Omicron), and G204R (Alpha, Gamma, and Omicron) as common mutations. And it’s also reported that R203K/G204R in N protein increased viral RNA binding ability.^146–149^

Hence, more evidence was required to reveal the mutational impact on the E, M, or N protein of the SARS-CoV-2 variants.^149^

### SARS-CoV-2 non-structural proteins

Nearly two-thirds of the genome coded ORF1a/ORF1ab can be translated into polyprotein pp1a and pp1ab. The translation of ORF1ab is realized by −1 frameshift at C13468, allowing continued translation instead of termination. The translated product pp1a/pp1ab could be proteolytically cleaved into non-structural proteins (Nsps) with different functions by viral proteases.^20,141,150,151^

**Fig. 4:**
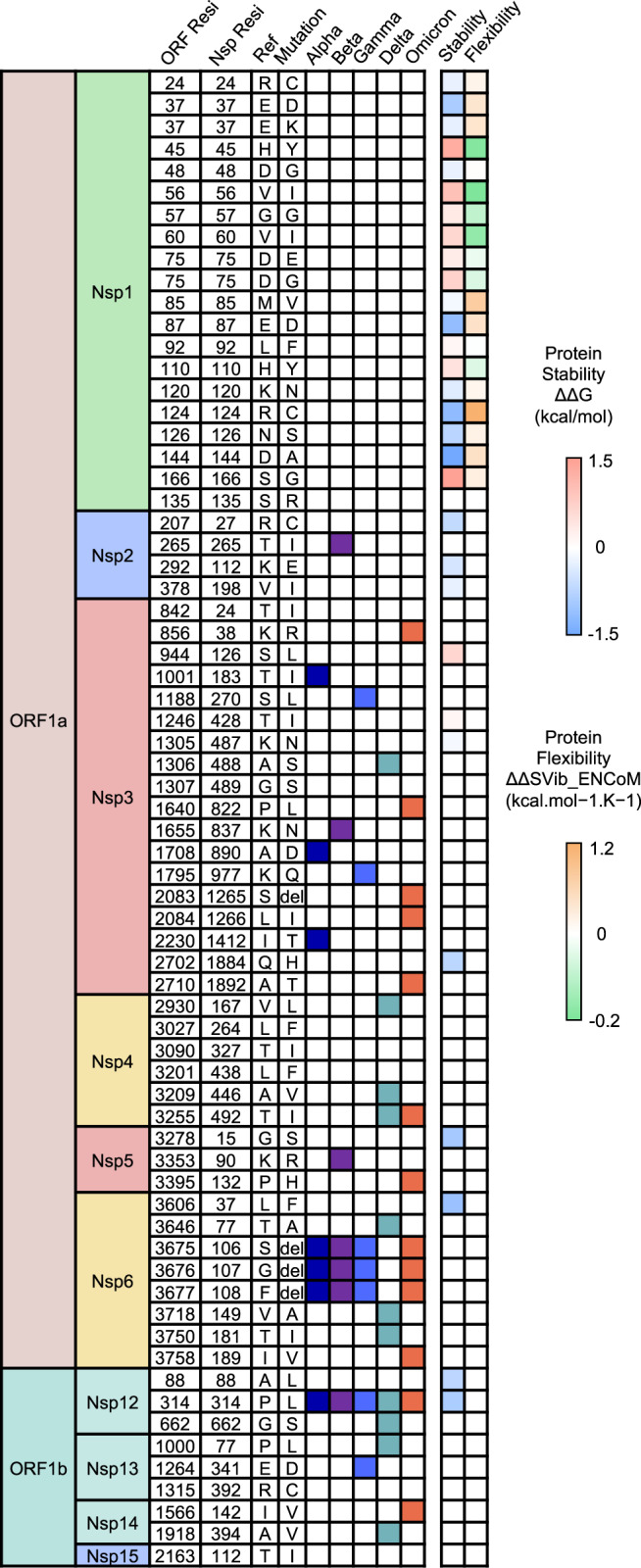
Heatmap of the mutation site and the mutational impact of SARS-CoV-2 variants on the non-structural proteins. The mutation site and its wild type and mutant residue in ORF sequence and detailed non-structural protein sequence among all variants are displayed. Each mutation is marked in a different color in the representation of the occurrence in VOCs and color-scale in reflection of the impact on protein stability and flexibility. Details for each mutation can be found in Supplementary Table 1

Among the Nsps, Nsp3, Nsp5, and Nsp12 received the most attention for both biological investigation and drug development, as Nsp3 and Nsp5 were viral protease PLpro (Papain-like protease)^152,153^ and Mpro/3CLpro (Main protease/3 chymotrypsin-like protease)^154,155^ mediating polyprotein cleavage and Nsp12 was the RNA-dependent RNA polymerase along with co-factor Nsp7/8 mediating genome replication and transcription.^156,157^ Therefore, mutations in these key viral proteins may further impact viral survival (Fig. 4). Nsp3 is now reported with the highest variation rate among the non-structural proteins and is closely related to the overall genome variation. Besides, great mutation diversity among the VOCs was observed since no shared mutation in Nsp3 was found among them. In comparison, relatively few mutations were found in the Nsp5 and Nsp12 (Nsp5:K90R-Beta, Nsp5: P132H-Omicron, Nsp12: P314L-All, Nsp12:G662S-Delta), and hence these two Nsps were recognized as relatively static in circulating variants. A mutational analysis using computational modeling is recently reported to reveal the influence of mutations in Nsps on protein stability and flexibility showed little mutation from VOCs with a significant impact on the Nsps, especially Nsp3, Nsp5, and Nsp12. Such as mutation P314L at Nsp12 was reported to decrease the protein stability.^27,45,61,134,158–163^

In summary, the evolution of SARS-CoV-2 endowed each clade with a distinct pattern of mutations. These mutations exerted various impacts on viral molecular characteristics. Current studies focused on investigating the mutational impact on spike protein due to its critical role in receptor binding and antibody evasion. It was broadly found that the mutations could alter the protein characteristics to benefit from ACE2 binding and diminish the binding with neutralization antibodies. In comparison, relatively less attention was paid to the mutational impact on other structural and non-structural proteins. Although sequential analysis has revealed mutations on these proteins from SARS-CoV-2 variants, little effort was given to the in-depth study of their influence on the protein structure and function and potential resistance to targeted drugs.^141,153^

### Immune evasion of SARS-CoV-2 variants from current therapeutic agents

#### Neutralizing antibodies

**Fig. 5:**
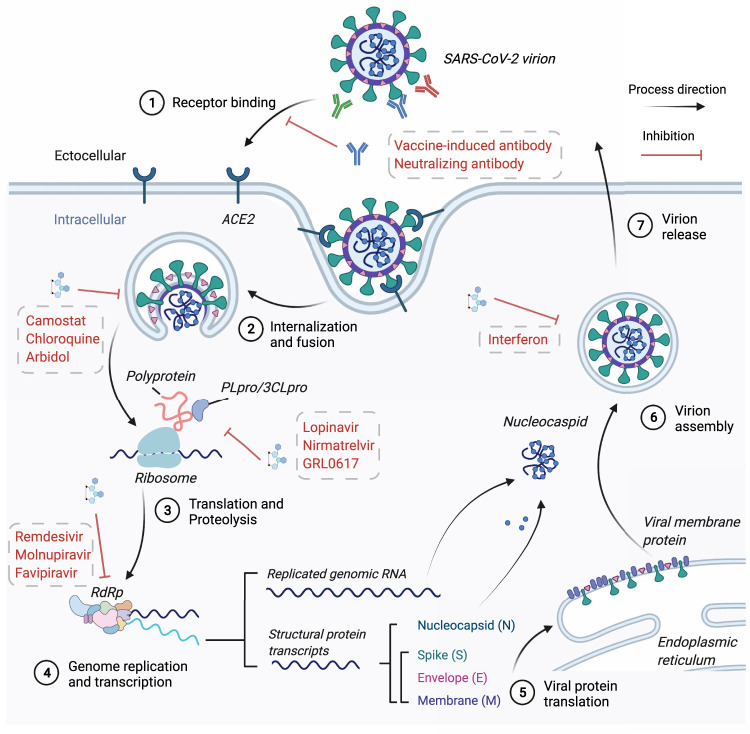
Overview of SARS-CoV-2 infection and replication and the mechanism for anti-SARS-CoV-2 therapeutic agents. Step1: spike protein of SARS-CoV-2 binds to the host ACE2 to initiate viral attachment and endocytosis. The vaccine-induced antibody, neutralizing antibody, and ACE2-mimic protein could block this step. Step2: spike protein was cleaved by host TMPRSS2 to mediate spike protein conformation and viral fusion with the host membrane in an acidic endosomal environment. Small molecules such as Arbidol acted in this step. Step3: the released viral RNA genome is translated into polyproteins, and the translated product undergoes proteolysis by viral protein PLpro and 3CLpro to generate mature non-structural proteins and initiate viral replication. Protease inhibitors such as Nirmatrelvir target this step. Step4: the assembly of RNA-dependent RNA polymerase (RdRp) complex would start viral genome replication and transcription for structural proteins. RdRp inhibitors such as Remdesivir target this step. Step5: the transcripts for structural proteins would be translated further into the cytoplasm for N protein and in the endoplasmic reticulum for S, M, and E proteins. Step6: replicated RNA genome binds with N proteins to form nucleocapsid, and it would further assemble with other structural proteins in the membrane envelope. Immune modulators such as interferon act in this step. Step7: the assembled and enveloped virion is transported to the cell membrane and released. BioRender was used to generate this figure

The preliminary step for viral entry was the spike protein binding to the host cell receptor ACE2 (Fig. 5). Neutralizing antibodies (nAbs) targeting the spike protein, especially the RBD domain directly located at the interface with ACE2, can neutralize viral infection by blocking receptor recognition.^164^ Moreover, nAbs could also mediate antibody-dependent cellular cytotoxicity (ADCC) to eliminate infected host cells expressing the spike protein.^165–168^ Thus, many neutralizing antibodies against spike protein were developed as potential therapeutic agents against acute SARS-CoV-2 viral infection.^169–171^

According to the binding epitopes, SARS-CoV-2 nAbs can be classified as RBD-targeted nAbs, NTD-targeted nAbs and other protein-targeted nAbs^172–177^ and RBD-targeted nAbs accounted for the most widely-accepted antibody type entering the clinical trials for preventing SARS-CoV-2 infection.^178–181^ Currently, nine neutralizing monoclonal antibodies and their combinatorial cocktail, Bamlanivimab (LY-CoV555),^182^ Etesevimab (LY-CoV016),^183^ Casirivimab (REGN10933), Imdevimab (REGN10987)^184,185^ Cilgavimab (AZD1061/COV2-2130), Tixagevimab (AZD8895/COV2-2196),^186^ Sotrovimab (VIR-7831),^187^ Regdanvimab (CT-P59)^188,189^ and Bebtelovimab (LY-CoV1404)^183^ targeting SARS-COV-2 spike protein were issued with emergency use authorization (EUA) by FDA for treatment of mild to moderate SARS-CoV-2 infected individuals. These authorized antibodies all displayed potent neutralizing capability against the ancestral strain of SARS-CoV-2.^5,164,190,191^

With the emergence of SARS-CoV-2 variants, many studies investigated the altered efficacy against viral infection of these antibodies.^4,192–198^ The development of approaches in studying antibody’s molecular and structural characteristics has provided preliminary insight into the changes of binding dynamics of spike-antibody and the following potential impact on antibody neutralization efficacy as described above.^197–200^ With multiple critical mutations destabilizing the antibody interaction with spike or RBD, in vitro experiments also revealed the worrying performance of current antibodies against the SARS-CoV-2 variants.^201,202^

**Fig. 6:**
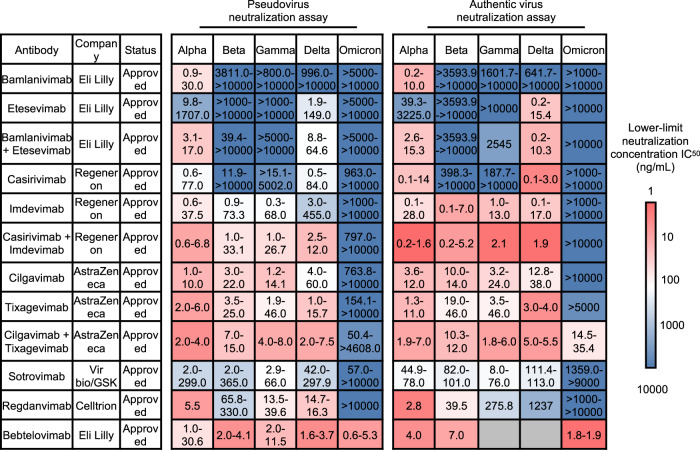
The summary of neutralization capability of approved antibodies against in vitro pseudovirus and authentic virus infection from SARS-CoV-2 variants. The ranges of reported neutralization capability against each VOC are listed. The lower-limit concentration IC_50_ are indicated in a red-to-blue color scale to represent the minimal neutralizing capability of each antibody reported. A bluer color indicates a reported stronger evasion of VOCs from antibody neutralization than ancestral strain. The detailed antibody neutralization concentration from each study could be referred in Supplementary Table 2

Considering that in vitro neutralization for the same therapeutic agent might differ under different circumstances, including virus types, cell culture types, and reference virus isolation, we listed the pseudovirus or authentic virus half-maximal inhibitory concentration (IC_50_) values of the mentioned authorized antibodies in range by reference to collective information from Stanford University Coronavirus Antiviral & Resistance Database and the contained corresponding studies on each antibody (Fig. 6).^203–205^

Most antibodies or cocktail pairs maintained the neutralizing efficacy against Alpha, Beta, Gamma, and Delta variants.^5,117,206^ Only Bamlanvimab showed great vulnerability toward all the VOCs, and the combination use of Etesevimab could barely improve the efficacy against specific strains.^5^ However, in terms of the Omicron strain, a significant reduction of neutralizing titer (>100 folds decrease) against both pseudovirus and authentic virus of all antibodies can be observed.^5^ In 2021, the use of Bamlanivimab alone was withdrawn from the authorization list due to its limited capability to block Beta and Gamma infection.^207^ Interestingly, the EUA of Bamlanivimab and Etesevimab in combination was later revised for post-exposure prophylaxis (prevention) because of their neutralizing potency against Delta, but the antibody cocktail received withdrawal again against Omicron for lack of effectiveness both in vitro and in vivo by November 2021.^208^

It should be noticed that an antibody pair, Cilgavimab+Tixagevimab (Evusheld) could maintain its efficacy against VOCs. Cilgavimab+ Tixagevimab showed a 5-folds to 12-folds decrease at a minimal neutralization against authentic Omicron, while the independent use in neutralizing assay displayed even significantly diminished efficacy (IC_50_ > 10,000/10,000 ng/mL in pseudovirus assay and >10,000/5000 ng/mL in authentic virus assay).^193^

In comparison, although individual use of Sotrovimab showed great vulnerability to the evasion of the Omicron variant,^5^ Sotrovimab even showed potency in controlling in vivo viral infection by ADCC and antibody-dependent cellular phagocytosis and cross-neutralizing capability against other sabecoronovirus,^165^ providing a possible mechanism for its use.

The alarming evasion of VOCs from currently approved antibodies urged novel antibody discovery or development. Recently Bebtelovimab (LY-CoV1404), a novel antibody with the latest approval for clinical use, displayed outstanding performance (IC_50_ = 0.003 μg/ml) in neutralizing Omicron pseudovirus and authentic virus, shedding light on the development of effective antibody therapy against Omicron variant.^122,205,209^ Besides NTD domain targeting, RBD-NTD dual-targeting or multi-spike variants-targeting antibodies were under development, and early findings showed their potential advantage in neutralizing more mutated variants such as Omicron previously reported antibody-based on screening the binding affinity with wild-type RBD or spike.^210–214^ Moreover, cocktail usage such as Cilgavimab+Tixagevimab provided a potential strategy of rationally using currently available antibodies to avoid the significant deficiency in neutralizing specific VOCs during individual application.^188,215^ Nevertheless, antibody-based therapy still confronts a huge challenge from the current Omicron pandemic. A new strategy to optimize the discovery and development of novel antibodies in adaptation to the mutational impact on neutralizing efficacy of emerging SARS-CoV-2 variants is urgently demanded, and systematic surveillance of antibody efficacy.

#### Vaccines

Currently, prophylactic vaccines remain the mainstay preventing SARS-COV 2 infection.^216^ According to the WHO COVID-19 vaccine tracker and landscape, there are currently 153 candidate vaccines under clinical development and 196 candidate vaccines under preclinical development worldwide,^217^ among which 19 vaccines have been authorized or fully approved in various countries, and some have been incorporated into national vaccination programs and widely applied.^218^ At present, authorized vaccines using three major platforms, mRNA, adenovirus, and inactivated virus, account for over 95% of vaccination doses around the world, including the two mRNA vaccines BNT162b2 (Pfizer/BioNTech) and mRNA-1273 (Moderna), one adenovirus vaccine AZD-1222 (AstraZeneca) and two inactivated vaccines BBIBP-CorV and CoronaVac (Sinovac).^219–221^ Approved vaccines have shown substantial efficacy in both in vitro neutralization and populational scale against the ancestral strain of SARS-CoV-2,^222^ but emerging evidence indicated significant immune escape of VOCs from the vaccine-induced protection.^59,117^^,223–225^

**Fig. 7:**
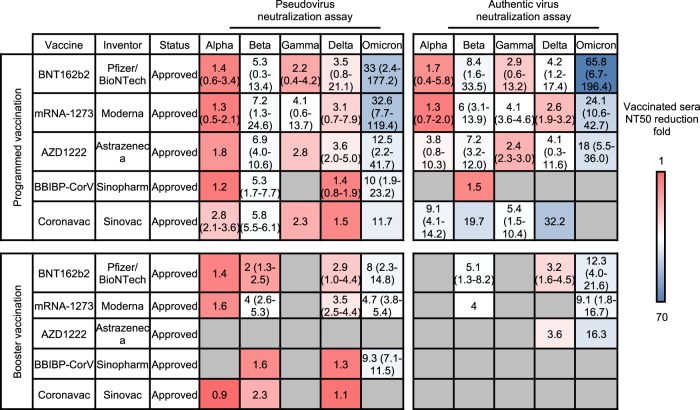
The summary of reduction folds of neutralization titers of elicited antibody by approved vaccines against in vitro pseudovirus and authentic virus infection from SARS-CoV-2 variants. The average and ranges of reduction folds of neutralization titers against each VOC compared to ancestral strain are listed. The average folds are indicated in a red-to-blue color scale to represent the average neutralizing capability of each vaccine against variants. A bluer color indicates a stronger evasion of VOCs from neutralization of vaccine-elicited antibodies in comparison to the ancestral strain. The detailed neutralization titer reduction from each study could be referred in Supplementary Table 3

Neutralizing antibodies induced by the antigen underlie the vaccine protection against SARS-COV 2 infection, and the neutralizing titer of serum from vaccinated people is a key indicator for evaluating vaccine effectiveness, making in vitro neutralization analysis a convenient but important method for monitoring vaccine-elicited humoral immunity against SARS-COV 2 variants.^226,227^ Nevertheless, due to the diversity in serum condition, experimental procedures, and statistical calculation for serum neutralization titer, current studies reported a wide range of 50% neutralization titer (NT50) of serum against each VOC strain. Therefore, we listed the reduction fold in NT50 of pseudovirus or authentic virus neutralization against each variant of the mentioned authorized vaccines in average and the range compared to the ancestral strain by reference to collective information from Stanford University Coronavirus Antiviral & Resistance Database (https://covdb.stanford.edu/) and the contained corresponding studies on each vaccine^228^ (Fig. 7).

In summary, sera from people receiving programmed or even booster vaccines showed a generally declined neutralization titers against different SARS-COV 2 VOCs compared with neutralization titers against the ancestral strain. For programmed vaccination, Alpha,^229–231^ Gamma,^232–234^ and Delta^235–237^ variants manifested relatively mild escape from neutralization (mostly ≤ 5 folds reduction), followed by the Beta variant^232,238,239^ (mostly 5–10 folds reduction). The most prominent reduction in the Omicron variant (mostly 10 folds reduction) was observed.^60,193,235,240^ This phenomenon was consistent with the evasion of therapeutic antibodies by Beta and Omicron variants discussed above, and neutralization assay results obtained from authentic virus and pseudovirus were generally parallel. The booster dose for each vaccine appeared to increase the neutralizing capacity of vaccine sera against VOCs by large, but the Omicron variants still presented obvious evasion from vaccine sera, with 8, 4.7, and 9.3-folds decrease for BNT162b2,^193,235,241–243^ mRNA-1273,^235,242,244^ and BBIBP-CorV,^118,245,246^ in the pseudovirus-based assay, and 12.3, 9.1 and 16.3-folds decrease respectively for BNT162b2,^4,60,242,247,248^ mRNA-1273,^242,247,248^ and AZD1222 in the authentic virus-based assay.^60^

Further insight into the Omicron variant evasion from neutralization showed an even remarkable decrease in vaccine protection over time. In a study, sera were obtained from 17 people in the second week, third month, and the sixth-month post BNT162b2 programmed immunization, and their neutralization against SARS-COV 2 wild type or Omicron variant was measured by authentic virus neutralization assay.^249^ It was found that the proportion of samples with neutralizing titer below the limit of detection against Omicron variants increased from 23.5% (2nd week) to 41.2% (3rd month) and 64.7% (6th month), suggesting that programmed vaccination of BNT162b could not elicit durable protection against the Omicron variant, and strengthening the necessity of booster vaccination.

SARS-CoV-2 VOCs, especially the Omicron variant, seriously affect vaccine-induced immunity from sera neutralization. Booster seems to be an ideal strategy for handling the immune evasion of the Delta variant but still partially compromises the Omicron variant. More importantly, as we have witnessed the continuing decrease in neutralizing efficacy post programmed vaccination, it deserves further observation on how well the neutralization titer post-booster could be maintained, and the time durability of elicited neutralizing antibodies could be another decisive factor influencing the final protective efficacy of vaccines.

#### Viral inhibitor drugs

Compared to the antibody, the viral inhibitor for SARS-CoV-2 focused more on the host factor facilitating viral infection and SARS-CoV-2 non-structural proteins.^205,250–253^ These proteins played a critical role during viral replication and maintained relatively sequential stability compared to the spike.^253–258^ Two mainstay strategies are adopted in developing antiviral drugs against COVID-19, including novel design of virus-targeted drugs and repurposing of currently available drugs with potential antiviral activity.^175,259,260^ However, it often took decades to develop novel drugs due to drug management authorities’ comprehensive pharmacological and biosafety evaluation. Hence, broad-spectrum drugs with prior validation against other pathogens such as viruses (SARS-CoV, MERS-CoV, HIV, EboV) and parasites (Malaria Plasmodium) received more attention for evaluation of their potential use in the ongoing pandemic, and these repurposed drug candidates are prone to large-scale manufacturing and delivery once sufficient evidence was acquired examining their effectiveness against SARS-CoV-2.^261–265^ Currently, various viral inhibitors are under clinical trial, and some have entered clinical use.^260,266–269^

The emergence of SARS-CoV-2 variants and broad reports of their evasion from neutralization by nAbs and vaccines lift the expectation of an anti-SARS-CoV-2 viral inhibitor.^270^ More and more experimental and clinical studies revealed the unique advantage of viral inhibitors in controlling the viral infection of VOCs due to the conservative drug target.^258,271–273^

**Fig. 8:**
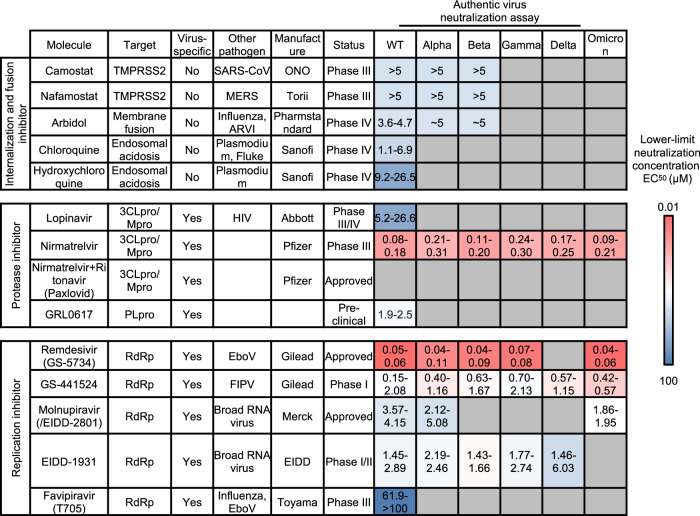
The summary of neutralization capability of small molecule drugs against in vitro authentic virus infection from SARS-CoV-2 variants. The ranges of neutralization capability of small molecule drugs targeting both viral and non-viral targets against VOCs are listed. The lower-limit concentration EC_50_ are indicated in a red-to-blue color scale to represent the minimal neutralizing capability of each antibody reported. A bluer color indicates a stronger evasion of VOCs from neutralization of small molecule drug in comparison to ancestral strain. The detailed small molecule neutralization capability from each study could be referred in Supplementary Table 4

The antiviral effects of small molecule inhibitors are determined by 50% and 90% effective concentration (EC_50_ and EC_90_) values. Here, we listed EC_50_ of small molecules in ranges with the reported value from corresponding studies on each molecule (Fig. 8).^274^

##### Broad-spectrum antiviral inhibitors

Various viruses, including influenza virus, SARS-CoV, and MERS-CoV, employ TMPRSS2 to cleavage the fusion protein to mediate viral-host membrane fusion. Thus, TMPRSS2 inhibitors have become a promising target for inhibiting virus infection.^275–277^ Camostat and Nafamostat mesylate are oral TMPRSS2 inhibitors, and both enter phase III clinical trials with previously reported applications on SARS-CoV and MERS-CoV infection.^278^ Furthermore, Nafamostat was reported superior to Camostat in specificity and effectiveness.^261,279^ Research reported that these two drugs could effectively block the virus infection of ancestral SARS-CoV-2.^261,275,276^ Moreover, another novel small molecule compound targeting TMPRSS2, N-0385, exerted equivalent potency against four VOCs, Alpha, Beta, Gamma, and Delta variants, with EC_50_ ranging from 2.1 to 13.9 nM from SARS-CoV-2 nucleocapsid staining assay, and 2.6–26.5 nM from dsRNA staining assay.^251,280^

Chloroquine is a widely used antimalaria and anti-autoimmune drug by modulating endosomal pH and disturbing the Clathrin-dependent endocytosis to inhibit pathogen entry into the host cell, for which it manifested broadly anti-pathogen activity.^281^ Chloroquine was first reported to be active against SARS-CoV-2 in vitro in early 2020, which promoted clinical trials and authorization.^264,274,282,283^ Later, it faded out of the list of antiviral agents against SARS-CoV-2 with disproved inhibiting infection at the cellular level and protection in clinical practice.^284–286^ Mpro/3CLpro and PLpro play essential roles in transforming viral polyprotein into an active form in SARS-COV-2 replication.^287–289^ Therefore, drugs targeting the two proteins may significantly reduce the viral replication in the host cell.^290–293^

Lopinavir was the firstly-reported 3CLpro inhibitor for SARS-CoV-2, repurposed from inhibiting human immunodeficiency virus 1 (HIV-1).^294^ It has entered Phase III/IV clinical trials (ClinicalTrials.gov number, NCT04738045, NCT04328285, NCT04364022) with preclinical support (estimated EC50 26.63 μM in vivo).^271^ However, Lopinavir-Ritonavir provided no benefit for severe Covid-19 patients and few studies investigate variants susceptibility to the drug.^295^ Later, Nirmatrelvir was raised by Pfizer as a novel 3CLpro inhibitor for SARS-CoV-2. The FDA approved the Paxlovid comprising Nirmatrelvir and another molecule Ritonavir, to postpone drug metabolism in vivo, with Emergency Use Authorization (EUA) in December 2021.^296,297^ Comprehensive studies were performed to investigate its efficacy against SARS-CoV-2 VOCs. Results indicated that Nirmatrelvir-maintained effective (EC_50_ 0.08–0.18 μM—WT and 0.09–0.21 μM—Omicron) against various VOCs, including Omicron. This can be explained by the conserved bind pattern between P132H 3CLpro in complex with Nirmatrelvir under structural simulation.^258,272,298,299^ GRL-0617 was reported to be a novel drug targeting the PLpro of SARS-CoV-2. Despite the status as a preclinical study, the relatively high efficacy against ancestral strain infection in vitro (EC_50_ 1.9–2.5 μM—WT) may suggest its bright future for further investigation.^300–302^ Studies also pointed out that combinatorial use of 3CLpro and Mpro inhibitors could significantly inhibit SARS-CoV-2 variants, broadening the use of drugs targeting these two proteins.^303,304^

##### RdRp inhibitor

RNA-dependent RNA polymerase (RdRp) is employed by RNA viruses, including SARS-CoV-2, to replicate the viral genome and translate the protein, and such critical functions make it a great target for drug design.^253,305^ Several RdRp inhibitors, repurposed from another pathogen usage, were approved or entered clinical trial as SARS-CoV-2 inhibitors, such as Remdesvir, GS-441524, Molnupiravir, EIDD-1931, and Favipiravir.^306^

Remdesivir, an adenosine analog created by Gilead as an Ebola virus inhibitor, was the first to show that it could bind SARS-CoV-2 RdRp and disrupt RNA replication, acting as a translocation barrier. Remdesivir was the first drug approved by FDA for COVID-19 in pediatric and adult hospitalized patients in May 2020.^262^ A parallel study manifested even stronger antiviral activity against all VOCs than Nirmatrelvir (EC_50_ 0.05–0.06 μM—WT and 0.04–0.06 μM—Omicron).^272^ Although in vitro experiments have suggested that it displays great resistance to VOCs’ mutation,^272^ a recent meta-analysis revealed that the medication has no effect on COVID-19 protection, and WHO announced a conditional recommendation against remdesivir’s use in hospitalized patients.^266,307^ These controversial results raised questions about whether Remdesivir was clinically effective in inhibiting SARS-CoV-2 emergent variants and whether the RdRp-targeted drugs were an ideal strategy to overcome the evasion of variants from therapeutic agents.^272^ The active metabolite of Remdesivir, GS-441524, entered the Phase I clinical trial. It was once used to inhibit the Feline infectious peritonitis virus (FIPV), a coronavirus, targeting the viral RdRp.^308,309^ GS-441524 was also effective for inhibiting all VOCs, with almost unaffected efficacy for the Omicron variant (EC_50_ 0.15-2.08 μM—WT and 0.42-0.57 μM—Omicron).^272^ Whereas EIDD-1931 and its active form Molnupiravir (EIDD-2801) is also a nucleoside analog developed by Drug Innovation Ventures from Emory University, also targeting the SARS-CoV-2 RdRp.^310,311^ Molnupiravir has been approved by FDA for clinical use as the first oral drug treating SARS-CoV-2, and it displayed broad efficacy against multiple RNA viruses.^270,294,312^ Studies found that it could effectively inhibit the infection of both SARS-CoV-2 ancestral strain and Omicron variant in vitro (EC_50_ 3.57–4.15 μM—WT and 1.86–1.95 μM—Omicron).^5,263^

The viral inhibitor developed for SARS-CoV-2 mostly manifested maintained efficacy against the VOCs, significantly different from the performance of currently approved antibodies or vaccines.^258,272,313^ Although non-viral specific drugs such as Camostat could theoretically avoid the mutational impact of SARS-CoV-2 variants due to the independence of viral target,^313–316^ their general antiviral capability against authentic virus infection in vitro were relatively inferior to the viral-specific drugs such as Nirmatrelvir.^258^ However, more evidence has shown that non-structural proteins were also under the pressure of viral mutations.^25,159^ Despite the outstanding efficacy of currently approved 3CLpro inhibitors like Paxlovid and RdRp inhibitors like Molnupiravir against the Omicron variant, more attention should be paid to the future possible strain with significant mutations located on these viral proteins affecting the drug efficacy.

### Impacts of SARS-CoV-2 variants on pandemic control

#### The epidemiological landscape of variants

The persistent pandemic has witnessed the epidemiological change in virus spread due to various factors, including the emergence of variants, application of vaccines, and disease control policy implemented by global society.^317–319^ Globally, over 450 million infection cases and 6 million death cases were reported by the WHO (February 2022). Europe and the Americas accounted for the most confirmed cases, followed by Asia, Oceania, and Africa. Each SARS-CoV-2 VOC strain superseded the previous one to become the regionally or globally dominant strain during the pandemic. All the VOCs manifested diverse transmission dynamics, responses to vaccines, and impacts on infection outcomes to the difference in molecular profiles and immune escape as we have described. Hence, the epidemiological characteristics of SARS-CoV-2 variants would provide the preliminary impression for the investigator to analyze their distinct behavior in the global spread and immune evasion.

**Fig. 9:**
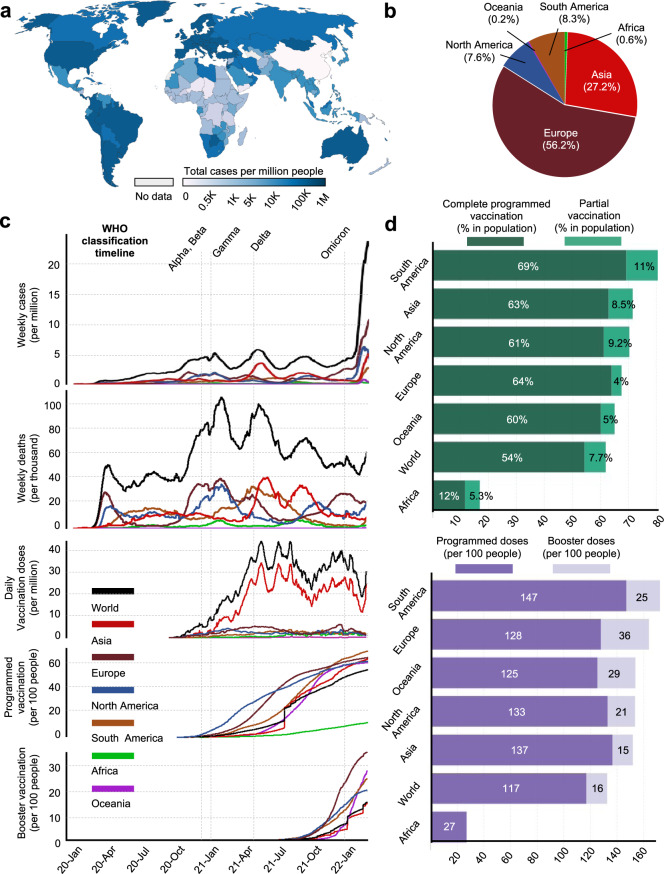
Epidemiological profile of SARS-CoV-2 infection and populational vaccination. **a** The global distribution of accumulated confirmed COVID-19 cases was reported by the WHO COVID-19 dashboard in February 2022. **b** The aligned trend of global weekly cases per million people, weekly death per million people, daily vaccination doses per million people, the proportion of programmed vaccination per 100 people, and proportion of booster vaccination per 100 people from January 2020 to February 2022. The timeline of emergent VOC by WHO classification is marked, and the regional data curve is colored differently. **c** The regional proportion of emerging cases of COVID-19 in February 2022. **d** The regional data of applied doses for programmed vaccination and booster vaccination. All the data comes from the website Our World in Data (https://ourworldindata.org/coronavirus), WHO COVID-19 dashboard (https://covid19.who.int/), and the figures in related are generated under the CC-BY 4.0 permission

We collected the data from Our World in Data^320^ and WHO COVID-19 dashboard^321^ from January 2020 to February 2022 and aligned the time scale to comparatively overview the influence of emerging SARS-CoV-2 VOCs (Fig. 9). The designating timepoint of each VOC was marked as a reference timeline for evaluation. It was shown that the emergence of all VOCs was closely correlated with an increased number of weekly reported infected cases and death cases, which also explained the meaning of “Variant of Concern”. In particular, the Omicron variant manifested astonishing capability in causing emergent infection, with a peak of over 20 million cases per week by the end of February 2022. It was yet to cause more death cases per week than Alpha, Beta Gamma, and Delta variants. Along with the emergence of VOCs, the worldwide application of vaccines also underwent steep expansion. It was shown that the early emergence of variant Alpha and Beta was associated with the initiation of global vaccination. Then, the emergence of the Gamma and Delta variants urged a more rapid application of vaccines around the world, and peak vaccination dose per day (around 40 million doses) could be observed right after the Delta variant was listed in the VOC. Despite that no emergent VOC was announced from June to October 2021, the global vaccination campaign maintained a relatively continuous trend until the emergence of the Omicron variant. With reports on the evasion of Delta, booster vaccination was started but still not widely applied. However, the significant evasion of novel SARS-CoV-2 variants from current antibodies and vaccines by prospective experiments revealed the deficiency of programmed vaccination, and it can be observed that the emergence of Omicron variants was highly associated with the rapid application of booster vaccination, indicating a global consensus on the necessity of improving vaccination efficacy against emergent SARS-CoV-2 variants.

However, the documented infection and vaccination status displayed a prominent imbalance between regions. Europe and the Americas recorded the most infection and death cases but received more doses for both programmed and booster vaccination in the population. The proportions of people with completed programmed vaccination in South America, North America, and Europe were 69%, 61%, and 64%, but the proportion of confirmed cases in the world of these regions was around 50%, 8%, and 8% by the end of February 2022. In comparison, new cases proportion (0.6%) and applied doses for programmed vaccination (12%) in Africa were largely behind the world level. Reasonable speculation about the low number of infected cases and vaccination doses should include that the backward economy and relatively low administrative ability in Africa hinder a broad test for COVID-19 positive cases and wide use of vaccination, which could make this region a black box for actual viral transmission and affection to people’ health and a potential reservoir for viral spread and evolution in the human population. This could also be the case in other countries lacking the broad COVID-19 testing capacity, as the COVID-19 tests per one million people were reported to be low in Asian countries like Afghanistan.

#### Vaccine efficacy

The observed huge reduction in the neutralizing efficacy of vaccine sera against SARS-CoV-2 variants raises the concern of vaccine efficacy in preventing both infections and, more importantly, severe diseases,^322,323^ for which clinical studies were performed to evaluate the performance of vaccines in protection from viral infection and virus-induced diseases. The vaccine efficacy or effectiveness (VE) was used to describe the proportionate reduction in cases with endpoint signs among the vaccinated group compared to an unvaccinated group. It is calculated as follows:

$${{{\mathrm{VE}}}} = \left( {1 - \frac{{{{{\mathrm{risk}}}}\,{{{\mathrm{among}}}}\,{{{\mathrm{vaccinated}}}}\,{{{\mathrm{groups}}}}}}{{{{{\mathrm{risk}}}}\,{{{\mathrm{among}}}}\,{{{\mathrm{unvaccinated}}}}\,{{{\mathrm{groups}}}}}}} \right) \times 100\% $$

**Fig. 10:**
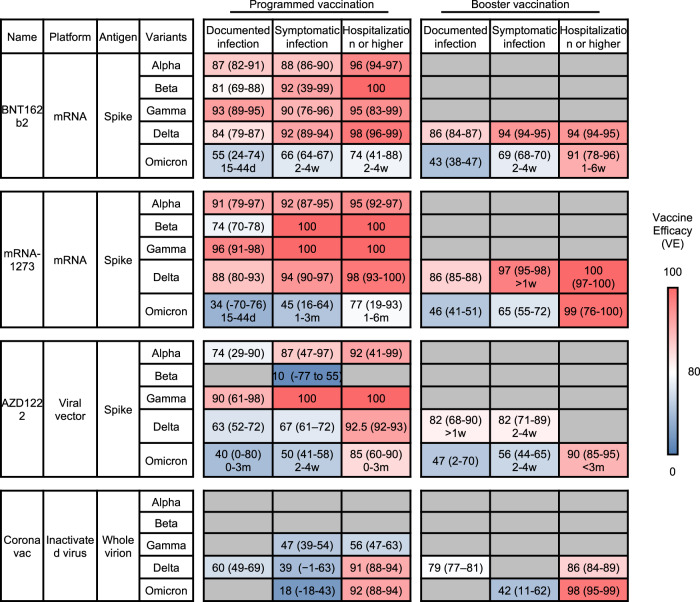
The summary of vaccine efficacy (VE) against infection from SARS-CoV-2 variants after programmed or booster vaccination. Three major outcomes are recorded, including documented infection, symptomatic infection, and hospitalization or more severe cases. Each vaccine’s average VE and 95% confidential interval (CI) against variants are listed. The average VE value is indicated in a red-to-blue color scale to represent the average protective efficacy against each outcome. A bluer color indicates worse protection from the outcome of vaccines. Detailed efficacy data could be referred in Supplementary Table 5

Three major endpoints were broadly used to evaluate vaccine efficacy,^324^ documented infection, symptomatic infection, and more severe hospitalization cases. The four vaccines, BNT162b2, mRNA-1273, AZD1222, and CoronaVac, were all effective against the ancestral strain of SARS-CoV-2, displaying high efficacy in preventing both asymptomatic and symptomatic infection^325,326^ but are now challenged by the emergence of VOCs. Here, we summarized the VE of four widely applied vaccines against SARS-CoV-2 VOCs and listed their performance in preventing different outcomes after programmed vaccination and booster vaccination (Fig. 10). Unless specified as d/w/m for the time post certain vaccination program, the VE reported in the table was calculated from 14 days post-vaccination until the study reached the research endpoint.

##### Programmed vaccination

The two mRNA vaccines, BNT162b2 and mRNA-1273, maintained equivalent protection efficacy against Alpha^327–331^ Beta,^221,330,332,333^ Gamma,^327,330,333,334^ and Delta^221,327,330,335–337^ variants in all major endpoints, with only a moderate decrease in protection against documented Beta variant infection.^338^ For the adenovirus vaccine AZD1222, VE for Beta variant symptomatic infection was found to drop dramatically to 10.4% (95% CI: −76.8 to 54.8) in a prospective study performed in South Africa among 2026 participants,^328^ while the protection against Alpha,^330,339,340^ Gamma,^330,334^ and Delta^339,341–343^ variant was mostly retained. This result was consistent with the observed decline in neutralizing activity of vaccine sera against the Beta variant as described above. For CoronaVac, data was comparatively limited.^344,345^ One large national cohort study in Chile, including about 2 million 6–16 years old participants, suggests that programmed vaccination of CoronaVac remains effective against Delta variant, reducing 74.5% (95% CI, 73.8–75.2) of symptomatic infection and 91.0% (95% CI, 87.8–93.4) of hospitalization.^346^ However, CoronaVac VE against Gamma variant 14 days post the second dose was estimated to be 46.8% (95% CI, 38.7–53.8) regarding symptomatic infection and 55.5% (95% CI, 46.5–62.9) regarding hospitalization, suggesting that Gamma variant may dramatically compromise CoronaVac-elicited immune protection.^341^

Vaccine protection against Omicron variant conferred by complete vaccination of BNT162b2, mRNA-1273, and AZD1222, significantly reduced. Programmed vaccination of AZD1222 was found to offer no significant protection against Omicron documented infection 14 days post-immunization in 2 recent studies,^347,348^ and only very limited protection against Omicron documented infection within 3 months post-immunization and VE against Omicron symptomatic infection or severe cases after vaccination was around 50% (2–4 weeks post) or 85% (0–3 months).^343^ For the 2 mRNA vaccines, VE against Omicron documented or symptomatic infection also decreased dramatically within 2-4 weeks after the programmed vaccination, while VE against hospitalization for Omicron maintained over 70% in the same period. A study investigating the recent Omicron outbreak in Hong Kong reported that programmed vaccination of CoronaVac and BNT162b2 offered minimal protection against mild/moderate disease but relatively robust protection (VE all over 70% in different age groups) against severe outcomes.^345^ In general, current evidence suggests that programmed vaccine protection, especially against documented or symptomatic infection, is substantially evaded by Omicron.

##### Booster vaccination

Since the clear reduction in VE of programmed vaccination was observed, a booster dose is now widely accepted, especially in countries with a high programmed vaccination implementation rate. VEs of booster vaccination listed in the table are calculated in people receiving homologous boosters compared to unvaccinated people. As discussed above, vaccine protection declined over time. Reports showed that VEs of 2 doses of mRNA vaccines (BNT162b2 or mRNA-1273) against documented Delta variant infection declined to less than 60% over 6 months, and the booster dose reinforced the VE to over 70%.^349^ In a recent study, VE against symptomatic Delta or Omicron infection was investigated, with booster vaccination taken into consideration.^343^ It revealed that VEs with AZD1222, BNT162b2, or mRNA-1273 against Omicron symptomatic infection reached approximately 48.9%, 65.5%, or 75.1% within 2–4 weeks post programmed vaccination but then continuously declined to a half from the peak after the 15–19 weeks for AZD1222 and 10–14 weeks for the mRNA vaccines, while VE against symptomatic infection of all three vaccines barely existed 20 weeks post programmed vaccination.

Furthermore, VEs of primary AZD1222 vaccination followed by homologous or heterologous mRNA vaccine boost, and primary mRNA vaccination followed by a homologous or heterologous mRNA vaccine boost were investigated. Booster vaccination effectively elevates VEs against Omicron symptomatic infection to an equivalent or even higher level than VEs post programmed vaccination. The importance of booster vaccination, especially for senior citizens, has also been well addressed in the current Hong Kong outbreak of Omicron, in which the third dose of BNT162b2 or CoronaVac conferred a 71.9% (95% CI: 25.1–89.5) or 96.6% (95% CI: 85.7–99.2) extra protection against severe or fatal COVID-19 to people over 80 years as compared with programmed vaccination.^345^

In general, despite the reduction in neutralizing titers of vaccine sera against various VOCs, programmed vaccination displayed great performance in protecting people from symptomatic and severe infection of VOCs, except Omicron. All currently approved vaccines in programmed doses did not manifest clear protection from documented infection of the Omicron variant, and more importantly, the efficacy would decrease over time. Application of booster vaccination enhances vaccine efficacy regardless of the booster vaccine type, reinforces the declined protection, and protects against severe infection of the Omicron variant. Nevertheless, it remained a question of how durable the VE was after booster vaccination. More importantly, booster vaccination did not provide extra protection against documented infection of the Omicron variant, which alarmed the global society about the necessity of strict policy for controlling the virus transmission.

#### Virus transmission in human and animal populations

Multiple studies have identified the increased affinity of SARS-CoV-2 spike protein or RBD with receptor ACE2, leading to a possible higher viral infectivity or faster transmission.^36,68,73,350–352^ The SARS-CoV-2 fitness is a concept to depict the advantages of certain strains during virus spreading and transmission, including stronger binding to ACE2-expressing cells, higher fusion activity, more rapid transmission between hosts, faster replication, and increased viral loads in infected subjects.^22,26,353–357^ Moreover, as the ACE2 of other mammals share sequence similarity with human ACE2, the mutations located on RBD that affect the binding with human ACE2 could increase the affinity to ACE2 of other mammals and increase their susceptibility to SARS-CoV-2.^357–364^

##### Human transmission

**Fig. 11:**
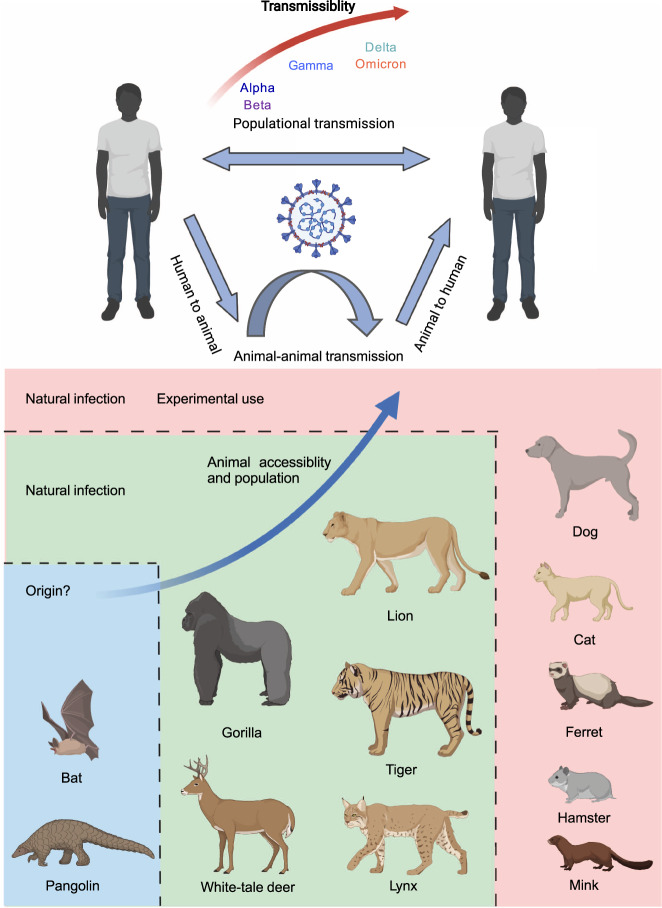
Change in SARS-CoV-2 variants transmissibility and patterns for transmission among human and animal populations. The transmissibility of SARS-CoV-2 VOCs increased over strains during human-to-human transmission. And substantial evidence reveals the SARS-CoV-2 transmission from humans to various mammalian animals as reported by OIE (https://www.oie.int/en/what-we-offer/emergency-and-resilience/covid-19/). There have been cases reporting the transmission between animal populations and animals to humans, making wild animals or pets a potential reservoir for viral preservation and evolution. BioRender is used to generate this figure

As multiple in vitro and in vivo research have identified the viral fitness change under the influence of certain mutations in the spike protein from different VOCs, the transmission efficiency in the population could be altered. For in vitro study, single mutation N501Y, D614G, L452R, and P681R and set mutations from Alpha, Delta, and Omicron in spike protein were found to increase the viral fitness, enhancing both transmissibility and replication, indicating that except evasion from neutralization, mutations of SARS-CoV-2 can bring in advantage for viral transmission.^56,206,365–370^ Hence, populational studies were also performed to investigate the transmission dynamics of SARS-CoV-2 variants (Fig. 11).

In general, epidemiologists have studied the change in transmissibility of each of the VOCs compared to the co-circulating VOCs at their time. Alpha, Beta, Gamma, and Delta variants, were reported with higher transmissibility of 43–90%,^371^ around 50%,^43^ 170–240%,^372^ or 130–170%^373^ than their co-circulating VOCs, respectively, and Omicron is estimated to be even more transmissible compared with Delta variant, especially in the vaccinated population.^40,371,374–379^ A recent study conducted in Denmark analyzed the transmission of Delta and Omicron variants among 11,937 households and found that Omicron was 2.61 (95% CI: 2.34–2.90) times or 3.66 (95% CI: 2.65–5.05) times more transmissible than Delta among fully-vaccinated households or booster-vaccinated households, but only merely 1.17 times (95% CI: 0.99–1.38) among unvaccinated people, suggesting that the real-world advantage of Omicron variant in transmission over Delta variant may be largely attributed to the evasion of vaccine-elicited protection of Omicron, especially in the context of broad vaccinated population.

##### Animal transmission

With increasing studies revealing the sequence similarity between human ACE2 and other mammalian ACE2, growing evidence indicated that SARS-CoV-2 can also infect animals.^360,363,364,380–383^ An international organization OIE (World Organization for Animal Health), recorded the investigation of animal infection of SARS-CoV-2. Over 600 outbreaks in animals have been reported worldwide, affecting 19 species in 35 countries (Fig. 7^384,385^). Currently, animals including the Feline family (Cat,^386–389^ Lion, Tiger,^390^ Snow leopard,^391^ Fishing, cat,^392^ Lynx), Dog,^358,393–395^ Mink,^111,389^ Otter,^396^ Ferret,^394,397,398^ Gorilla, Deer,^399–402^ Binturong, Coatimundi, Hippo,^403^ and Hamster^404,405^ were reported with positive cases of SARS-CoV-2 infection. These results suggested the existence of a human-to-animal transmission pathway.

Moreover, evidence shows animal-to-animal transmission between cats, minks, ferrets, and hamsters, which even shows that the animal population near human activity could be a repository for SARS-CoV-2.^394,404,406–408^ Studies on mink farms indicated that SARS-CoV-2 could transmit between human and mink and back to human,^111,408^ while non-synonymous mutations could be found in the mink sequence. These results further implied that SARS-CoV-2 evolution could occur during intra-animal populational transmission, and such mutant strain could be transmitted back to humans by animal-to-human transmission.

Besides the study on the general transmission among animals, SARS-CoV-2 variants manifested an inclination of broad host spectrum tropism. In vitro studies have shown that various VOCs displayed higher affinity to murine ACE2, and mice challenged with the authentic virus of Alpha variant developed pathological changes along the respiratory tract compared to the ancestral strain.^409^ The primary outcomes suggested that the host tropism of SARS-CoV-2 variants in animals tends to expand.

Generally, SARS-CoV-2 variants manifested an increase in transmissibility among the human population,^410–412^ and the Omicron variant, with its remarkable evasion from neutralization, displayed an even stronger populational prevalence.^374,413^ Furthermore, the findings of animal infection of SARS-CoV-2 further displayed that the virus enjoyed broader transmission due to the wider host tropism and huger reservoir for viral evolution, which could facilitate the emergence of SARS-CoV-2 variants and exacerbate the burden of global cost in containing the pandemic. More evidence was required to demonstrate an enhanced transmission of SARS-CoV-2 variants to animal populations in wild conditions.

#### Clinical presentation and complications

With the broader distribution, enhanced evasion, and improved transmissibility mentioned above, SARS-CoV-2 variants infection cause more heterogeneous outcomes in patients mainly in two ways: the stronger but comprehensive ability to cause severe diseases due to immune escape from host immunity and faster replication, or the strain-specific mutational impact on viral protein leading to diversity in pathogenesis.

##### Overall clinical outcome

Risks of hospitalization and severe cases of death related to Alpha,^378,414^ Beta,^414^ Gamma,^414,415^ or Delta^416^ variant infections increased. Conversely, the Omicron variant shows a decrease in disease severity. This is consistent with the laboratory finding that Omicron infected mice showed reduced replication in respiratory tracts and ameliorated lung pathology compared with ancestral strain or Delta variant infected mice, and weight loss and mortality rate of Omicron infected mice were also lowest.^417^ Epidemiologically, Omicron variant infection was associated with a lower risk of hospitalization, ICU admission, mechanical ventilation, and a shorter length of hospital stay than Delta variant infection by large.^414,418^

##### Acute clinical presentations in common

Clinically, the infection of SARS-CoV-2 is diagnosed with reverse-transcription PCR as the gold standard and could be classified into different clinical types according to clinical manifestations and radiological examinations.^419^ Severe COVID-19 in adults is defined as meeting any of the following conditions: dyspnea with the respiratory rate of 30/min, blood oxygen saturation of 93%, the ratio of the partial pressure of arterial oxygen to the fraction of inspired oxygen (Pao2:Fio2) 300 mm Hg, or lung imaging showing infiltrates in more than 50% of the field.^420^ In a large case series published by the Chinese Center for Disease Control and Prevention early in the pandemic, mild, severe, and critical cases accounted for 81%, 14%, and 5%, respectively, and the case-fatality rate was 2.3% in the 44,672 confirmed COVID-19 cases series.^421^ Nevertheless, severe cases and mortality rates vary as the vaccination campaign progresses. Pandemic lineage shifts variant and experience in treatment accumulates, which should be noticed in the following discussion about clinical manifestations of SARS-COV-2 infection, references of which would inevitably be limited by the representativeness of the study population and time.

In clinical practice, the average incubation period (interval between exposure to symptom onset) is approximately 5 days, and most people develop symptoms within 11.5 days after infection.^422^ Common symptoms include fever, dry cough, fatigue, and shortness of breath.^423,424^ Among hospitalized patients, common symptoms were fever (>90%), dry cough (60–86%), shortness of breath (53–80%), and fatigue (23–70%) (Fig. 8).^425–427^ In patients with mild symptomatic infection, fatigue, cough, and fever were reported with occurrence rates of 68%, 60%, and 56% as most frequent symptoms, and an altered sense of smell or taste was reported at a rate of around 3%.^428^ Besides these symptoms, sore throat, rhinorrhea, diarrhea, nausea, abdominal pain, myalgia, chest pain, dizziness, headache, anosmia, ageusia, testicle pain, and many other symptoms have been reported for SARS-COV-2 infection.^429–431^ As for laboratory findings, common laboratory abnormalities among hospitalized patients include lymphocytopenia (83.2%), thrombocytopenia (36.2%), and leukopenia (33.7%). Most patients had elevated C-reactive protein levels, and some had additional increased levels of aspartate aminotransferase, alanine aminotransferase, creatine kinase, or D-dimer.^425,432^ Chest radiographs or CT scans found that radiological abnormalities were common among hospitalized patients on admission, including consolidation (59%), ground-glass opacity (71%), and bilateral pulmonary infiltration (75%).^427^

**Fig. 12:**
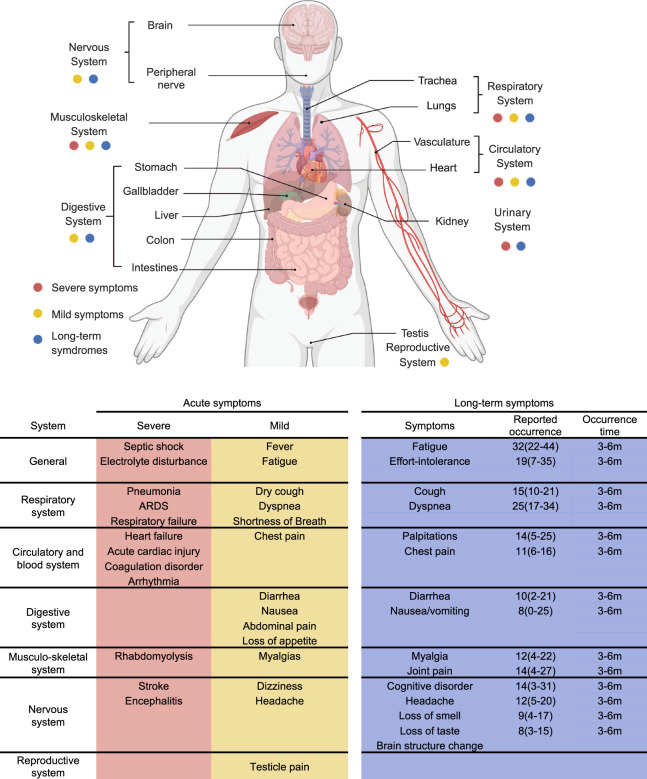
The overview of SARS-CoV-2 infection affecting the human system and summary of acute and long-term symptoms post. COVID-19 could lead to various symptoms occurring in various body systems except for the expiratory system. The summary table lists the clinical presentations of COVID-19, including severe and mild symptoms during acute infection and long-term symptoms with the occurrence time and rate. The reported occurrence rates of long-term symptoms are average with the 95% confidential interval (CI), and the occurrence time is presented as the time (m - month) post-infection. BioRender is used to generate the human body system presenetation

Infected patients could develop more severe complications,^433^ especially with risk factors including older age, comorbidities, immunocompromise, obesity, and heavy smoke.^433–436^ Most hospitalized patients (91.1%) are diagnosed with pneumonia by physicians on hospital admission, with a mean incubation of 3 days after onset of symptoms, and 3.4–8% of hospitalized patients developed ARDS (acute respiratory distress syndrome^426^). Extrapulmonary complications were observed in different organs and systems, including myocarditis,^437–439^ arrhythmia,^440–442^ myocardial ischemia^443–445^ regarding the cardiovascular system, acute kidney injury^446–448^ and electrolyte abnormalities,^449–451^ hyperglycemia,^452^ and ketoacidosis^453^ in the urinary system, endocrine system, stroke,^454–456^ and encephalitis^423,457,458^ regarding the neurological system (Fig. 12).^459^

##### Long-term post-acute clinical presentations in common

Besides acute symptoms and complications, SARS-CoV-2 infection could leave a long-lasting or fluctuating impact on patients, termed long COVID.^460,461^ The World Health Organization has defined it as a condition that “occurs in individuals with a history of probable or confirmed SARS-CoV-2 infection, usually 3 months from the onset of COVID-19 with symptoms lasting at least 2 months and cannot be explained by an alternative diagnosis.” It should be noticed that, for practical reasons, many studies investigating post-acute COVID-19 syndrome were based on self-report data, and the claimed symptoms were not validated with a comprehensive inspection to exclude another possible diagnosis, which means that they should not be recognized equivalently as long COVID.^462^ One important finding is that the post-acute COVID-19 syndrome is not just limited to patients with severe COVID-19.^463,464^ In a study investigating post-acute syndromes in SARS-CoV-2 symptomatic infection patients over 18 years old, 35% of the 274 symptomatic respondents, with over half of them under 50 years old, reported “not having returned to their usual state of health 2 weeks or more after testing”, and the most common symptoms were fatigue (71%), cough (61%), and headache (61%). Another study included self-reported data of around 100 thousand people with diagnosed COVID-19 infection previously and found that 37.7% of them reported at least one persistent symptom lasting for at least 12 weeks, with fatigue, shortness of breath, myalgia, and insomnia being the most common symptoms.^465,466^ A meta-analysis analyzed the prevalence of post-acute COVID-19 syndrome symptoms and found that many COVID-19 patients experienced long-lasting post-acute COVID-19 syndrome after recovery from the acute phase of infection.^467^ The clinical manifestations involve a wide range of systems, and the most common symptoms were fatigue (32%), dyspnea (25%), sleeping disorder (24%), and difficulty in concentrating (22%) within 3–6 months following infection. Many other symptoms, including depression, anxiety, palpitations, effort intolerance, chest pain, diarrhea, joint pain, myalgia, cognitive disorder, headache, and cough, are also found in no less than 10% of convalescent patients in this period. Under the current pandemic of the Omicron variant, infections continuously occur for evasion of vaccine and infection-induced immunity, meaning that a large proportion of people may suffer the post-acute COVID-19 syndrome, which raised the importance of further studies exploring underlying mechanisms and treatment of the post-acute COVID-19 syndrome^468^ (Fig. 12).

Especially a recent study based on UK Biobank data found that participants infected with SARS-CoV-2 showed a greater reduction in grey matter thickness and global brain size than the controls, with the reduction still being significant after excluding hospitalized cases.^469^ This study raised concern about the impact of SARS-CoV-2 infection on the neurological system.

##### Diversity in clinical presentation among VOCs

Evidence regarding the impacts of SARS-CoV-2 variants on certain symptoms was relatively limited. A previous meta-analysis found that anosmia was much more prevalent among populations predominantly infected with the G614 virus (pooled prevalence of 31.8%) as compared with populations predominantly infected with the D614 virus (pooled anosmia prevalence of 5.3%), suggesting that the D614G mutation contributed to the prevalence of anosmia in COVID-19.^470^ The Omicron variant was linked to a decrease in disease severity, which has been discussed above and was also found to impact clinical manifestation.^414^ Compared to Delta variant, anosmia was reported less often in Omicron variant infection cases (13% of Omicron cases, 34% of Delta cases, OR: 0.22, 95% CI: 0.21–0.23), and sore throat was reported more often in Omicron variant infection cases (53% of Omicron cases, 34% of Delta cases, OR: 1.93, 95% CI: 1.88–1.98).^471^ A Laboratory study found that replication of the Omicron variant was similar to the Delta variant in human nasal cultures but lower in lung cells and gut cells.^472^

With a sharp increase in infected cases worldwide, even mild cases of infection could be extremely troublesome for regions with poor medical resources. A variety of COVID-19 acute infection symptoms could affect almost every critical body system in humans.^459^ More serious consideration should be given to a wider and more rapid application of booster vaccination globally. The long COVID further suggests that the infection of SARS-CoV-2, even if not deadly, may bring a long-term negative influence on the infected population and decrease the life quality post-infection.^473^

#### Discussion and perspective

As the COVID-19 pandemic persisted, various SARS-CoV-2 variants emerged and became a major threat to public health.^110,474–476^ These variants harbored critical mutations in structural and non-structural proteins, affecting protein stability, antigenicity, and function.^317,477^ The accumulated impact on viral proteins at the single-residue level led to significant changes in biological behaviors of the virus, including infection, transmission, replication, and response to host immunity, and finally influenced the clinical phenotypes and presentations post-viral infection. Therefore, systematic studies connecting the molecular, biological, epidemiological, and clinical evidence of SARS-CoV-2 variants would be greatly demanded to provide insightful and constructive ideas for fundamental research on the virus itself and pandemic disease control.

The sequencing data from SARS-CoV-2 isolated samples provided evolutionary trace and revealed a newly-emergent strain. The evolutionary and sequential abundance analysis witnessed the Omicron strain as the most-diverged strain from the ancestors and currently the most prevalent strain. As more mutations were found, the greater concern was given to the surveillance of mutational impact on the critical viral proteins.^478^ Among the structural proteins of SARS-CoV-2, spike protein was regarded as the most important target determining the fate of viral recognition and fusion due to its binding with host receptor ACE2, for which mutation located on the spike protein exerted versatile influence on the structural characteristics, including receptor affinity, antibody binding, structural stability, and protein yield.^479^ These factors had an enormous and direct impact on the viral activity and response to host immunity and highlighted the value of current effort in closely monitoring the critical mutations leading to a significant alteration in vitro structural and biological features of spike protein from variants.^480^ Whereas, less attention was given to the accumulated mutations in non-structural proteins with extremely important biological functions during the virus life cycle, as they were by the large recognized stable invariants. However, the discovered mutation locus from the variants of concern at non-structural proteins with reported evidence showing the impact on structural stability doubts their “uninfluenced” prospect as a target for drug development. More experimental evidence was required to reveal the mutational impact on these key viral proteins in modeling the structural characters and mediating viral replication.

Since the mutations brought new molecular characteristics to key proteins of SARS-CoV-2, therapeutic strategies against viral infection confronted more challenges. It has been widely reported that all the VOCs manifested varied immune escape, especially the Omicron.^50,323,481,482^ Extensive effort has been put into revealing both molecular and immunological basis of the resistance of these variants to an antibody or vaccine sera targeting spike protein, and increasing evidence has indicated the connection between the structural change in protein-antibody complex and the diminished neutralizing capability of antibodies.^483–486^ These results possibly suggested a positive selection of emergent strains harboring mutations in spike protein with a potent immune escape from humoral immunity in the global population. The breakthrough of currently available recombinant or vaccine-induced antibodies has led to growing worried about the future development of antibody-based therapy.^190,322,487^ In comparison, recently, good news came from the small molecule drug targeting non-structural proteins such as 3CL protease inhibitor Nirmatrelvir and RdRp inhibitor Molnupiravir, as they manifested well-maintained antiviral activity against SARS-CoV-2 variants including Omicron during in vitro experiment. Therefore, closer monitor of drug resistance due to mutational change in non-structural protein targets and more high-level clinical evidence of drug efficacy are in demand to provide clear guidance in the use of anti-SARS-CoV-2 drugs against variants,^488–490^ while a “game-changer” method for developing variant-effective antibody is under great expectation.

With the changes in response to current therapeutic agents, SARS-CoV-2 variants exhibited various epidemiological and clinical manifestations differed by strain.^425^ The global epidemiological profile has shown an unprecedentedly rapid spread of the Omicron variant. Interestingly, global vaccination against SARS-CoV-2 rapidly increased since the first identification of the Alpha variant as VOC. In particular, the distribution of emerging infection and vaccination quota displayed a huge geographical imbalance and a mismatched relationship. Moreover, the vaccination efficacy confronted great challenges from the SARS-CoV-2 variants. Clear breakthrough has been observed among all vaccines for their programmed vaccination, and booster vaccination cannot provide significant sterile immunity toward the infection but higher efficacy in preventing symptomatic and severe cases. However, the duration of protection from the booster vaccination could be a key point for its efficacy against SARS-CoV-2, and more studies are required to answer this question.^491,492^ Besides, the higher transmissibility among the human population and wider host tropism of SARS-CoV-2 urged more attention to the transmission dynamics of the virus between humans or between humans and animals.^381,492^ As the potential virus bank, animal infection of SARS-CoV-2 might become an “Achilles’ heel” for disease surveillance and containment.^493^ Although an overall reduction in the Omicron variant-related death rate was observed, the increasing number of infected cases raised to worry about the global medical resources for curing the symptomatic infection. As more evidence has demonstrated the acute and long-term impact of SARS-CoV-2 infection,^420,443,494–497^ greater effort should be given to reduce populational infection instead of merely focusing on the number of death cases. Governments should realize the importance of collaboration in COVID-19 disease control.

## Supplementary information

Supplement table 1

Supplement table 2

Supplement table 3

Supplement table 4

Supplement table 5
